# Advances in Enhancing the Wear Performance of Ti-6Al-4V Biomedical Alloy Through Nb_2_O_5_ Coating

**DOI:** 10.3390/ma18071593

**Published:** 2025-04-01

**Authors:** Murilo Oliveira Alves Ferreira, Witor Wolf, Rogério Valentim Gelamo, Natália Bueno Leite Slade, Rodrigo Galo, Renato Goulart Jasinevicius, Carlos Alberto Fortulan, Jéferson Aparecido Moreto

**Affiliations:** 1 Materials Engineering Department, São Carlos School of Engineering, University of São Paulo (USP), São Carlos 13563120, SP, Brazilwitorw@usp.br (W.W.); 2 Institute of Technological and Exact Sciences, Federal University of Triângulo Mineiro (UFTM), Uberaba 38064200, MG, Brazil; rogelamo@gmail.com; 3 Institute of Exact Sciences, Naturals and Education, Federal University of Triângulo Mineiro (UFTM), Avenida Doutor Randolfo Borges Júnior, Univerdecidade, Uberaba 38064200, MG, Brazil; 4 Department of Dental Materials and Prosthesis, School of Dentistry of Ribeirão Preto, University of São Paulo (USP), Ribeirão Preto 14040904, SP, Brazil; rogalo@forp.usp.br; 5 Mechanical Engineering Department, São Carlos School of Engineering, University of São Paulo (USP), São Carlos 13566590, SP, Brazil; renatogj@sc.usp.br (R.G.J.);

**Keywords:** niobium pentoxide, reactive sputtering, surface modification, wear resistance, biomedical applications

## Abstract

The Ti-6Al-4V alloy is widely used in orthopedic and dental implants due to its excellent mechanical, corrosion, and biological properties. However, it exhibits several limitations that can compromise its performance in clinical applications. Notably, the alloy suffers from a high coefficient of friction, which can lead to increased wear and reduced longevity of implants under relative movement conditions. Additionally, Ti-6Al-4V shows susceptibility to localized corrosion in physiological environments, particularly in the presence of bodily fluids that may result in the formation of pitting. These challenges underscore the need for surface modifications that can enhance the alloy’s tribological performance, thereby improving its overall efficacy and durability as a biomaterial in medical settings. In this context, the manuscript presents applied and innovative research that assesses the impact of implementing nanostructured Nb_2_O_5_ coatings through the reactive sputtering technique on the wear performance of Ti-6Al-4V alloy under both air and artificial saliva (AS) solution conditions using a Pin-on-Disk apparatus. The nanostructured Nb_2_O_5_ coating demonstrated the ability to reduce the wear rate and volume by up to 88% without inducing any modifications to the R_a_ and R_t_ of Ti-6Al-4V, a feature that is desirable for applications in implantable devices. The reduction in wear can be attributed to the shift from adhesive wear mechanisms on uncoated surfaces to abrasive mechanisms on coated surfaces. This research highlights the strategic advantage of utilizing Brazil’s abundant niobium resources to advance biomaterial technology and facilitate applications that benefit public health.

## 1. Introduction

Applications of Ti-6Al-4V alloy are diverse, spanning various sectors that leverage its unique combination of high strength-to-weight ratio, excellent corrosion resistance, and biocompatibility [1]. Considering the biomedical sector, Ti-6Al-4V alloy may be used as orthopedic implants such as hip replacements, knee replacements, bone plates, dental implants, and other medical devices requiring biocompatibility and corrosion resistance in the body. However, as reported in the literature [2], in this specific field, Ti-6Al-4V alloy exhibits poor tribological properties, characterized by a high coefficient of friction (COF), low surface hardness, as well as high adhesive contact between the surfaces, which may be harmful to the human body. The exceptional corrosion resistance of Ti-6Al-4V alloy is primarily attributed to the spontaneous formation of a protective TiO_2_ passive oxide layer upon exposure to atmospheric conditions. This layer acts as a physical barrier, inhibiting uniform and localized corrosion processes. However, the integrity of this passive layer can be compromised in the presence of specific aggressive ionic species present in various corrosive environments and, mainly, in the physiological environment [3]. This compromise can lead to the initiation of localized corrosion processes, such as pitting, potentially degrading the mechanical integrity and biocompatibility of the implant material [4,5]. In this regard, the development of innovative and applied studies aimed at the surface modification of the Ti-6Al-4V alloy is of utmost importance, with the objective of enhancing its mechanical, anticorrosive, and biofunctional properties [5].

Numerous studies have been reported in the literature that consider the influence of surface modification on the Ti-6Al-4V alloy [6]. Ma Mohin et al. [7] improved the corrosion and cavitation erosion resistance of laser-based powder bed fusion-produced Ti-6Al-4V alloy by pulsed magnetic field treatment in saline and deionized water solutions. In addition, as noted by the authors, the surface treatment facilitated the formation of finely refined and uniform precipitates, resulting in a significant enhancement of the microhardness properties. Another recent and highly cited study was presented by Etrat Anees et al. [8], in which the authors used thin films of CH-xTiO_2_ (x = Ag, Mg, Sr, and Zn) composites deposited on the surface of the Ti-6Al-4V alloy via the sol-gel dip coating technique, with the aim of enhancing its applicability in dentistry. The results indicate that the CH-xTiO_2_ coating significantly enhances the corrosion resistance of titanium alloy surfaces when exposed to AS, thereby improving the durability and performance of dental implants. As indicated by the authors, considering the electrochemical impedance spectroscopy (EIS) measurements conducted in the presence of AS solution, the produced coating demonstrated a corrosion inhibition efficiency of up to 98%. The electrophoretic deposition of novel antibacterial and biocompatible polydopamine and ZIF-8 hybrid composite coating on anodized Ti-6Al-4V alloy with silane primary substrate was presented by Peyghan and colleagues [9]. Their findings indicate that the coating is an effective material for the reduction of *S. mutans* and *E. coli*, which constitutes another critical criterion for dental implant materials.

Sidra Sadaf Nisar and Han-Cheol Choe [10] investigated the mechanical, corrosion, and bioactive characteristics of TiO_2_ coatings doped with varying amounts of MoS_2_ nanoparticles, fabricated using the plasma electrolytic oxidation (PEO) technique on the surface of Ti-6Al-4V alloy. In comparison to pure TiO_2_, the TiO_2_ coating doped with MoS_2_ exhibited enhanced electrochemical corrosion resistance and improved cell proliferation properties. Considering the biological tests, alizarin staining, alkaline phosphatase activity (ALP), and mRNA expression analyses indicated that lower MoS_2_ contents, specifically P–2MoS_2_ and P–6MoS_2_, demonstrated superior cellular differentiation properties. It is worth noting that, despite the amount of treatment, the properties of the Ti-6l-4V alloy were improved. A novel method for creating a hardened surface enriched with TiN using nitrogen gas-assisted electrical discharge machining (NA-EDM) on the Ti-6Al-4V alloy was presented by Zaichao Liu et al. [11]. The NA-EDM-treated surface demonstrated enhanced resistance to wear and corrosion, exhibiting a notable 14.9% reduction in wear rate as well as in the average coefficient of friction (COF). In recent decades, a surface treatment technique that has gained prominence for enhancing the corrosion and wear performance of metallic materials is plasma-assisted physical vapor deposition (PVD) [12,13,14,15,16]. This method enables the production of coatings with minimal thickness, on the order of nanometers, which can be meticulously engineered to impart desired properties for various applications without compromising surface morphology [17,18,19,20,21,22]. One sector that benefits significantly from this characteristic is the biomedical field, where surface properties and the finish directly affect the acceptance and longevity of implantable devices. The PVD technique has proven effective in producing TiN and TiAlN/TiAlCrN coatings on the surfaces of the Ti-6Al-4V alloy, decreasing the corrosion current (i_corr_) and increasing impedance moduli values when compared to the base material [20]. As demonstrated in the preceding paragraphs, the application of coatings constitutes an effective strategy to enhance resistance to both uniform and localized corrosion processes, increase wear resistance, and improve the biofunctional properties of the Ti-6Al-4V alloy [23,24,25,26,27,28,29].

In recent years, our research group has focused on the production of nanostructured Nb_2_O_5_ coatings through the reactive sputtering technique, achieving notable results with Ti-6Al-4V alloy, 2xxx and 7xxx series aluminium alloys, and 316L stainless steel (316L SS) implantable devices [5]. The characteristics of the films produced by our group are distinctive, particularly concerning corrosion resistance (acting as a compact and protective barrier), mechanical properties (increasing the wear and fatigue crack growth resistance), and biofunctional properties (enhancing biocompatibility and reducing inflammatory response). Regarding wear performance, previous studies demonstrated the effectiveness of Nb_2_O_5_ coatings in reducing the wear volume for 316L SS, considering short immersion times in NaCl and AS solution [30,31]. Moreover, it is worth mentioning that a variation in temperature during the PVD deposition process led to a modification in the coating’s final performance. When considering a temperature of 25 °C for the sputtering process and comparing the uncoated and coated 316L SS specimens, the modification in the wear volume and wear rate reached 50% in the air environment [30]. Furthermore, despite not presenting an apparent reduction in wear volume after immersion in a 0.9 wt% NaCl solution, the Nb_2_O_5_ coating remained intact on the surface of the 316L SS, indicating that the substrate was not degraded and, consequently, showed an improvement in its wear performance when compared to the uncoated material. At a temperature of 300 °C, the reduction in the wear rate reached up to 90% in comparison to the uncoated 316L SS [31]. Additionally, the same effect of maintaining the integrity of the coating inside the wear track for all environmental conditions analyzed was obtained and proved by scanning electron microscopy (SEM) combined with energy dispersive X-ray analysis (EDX) analysis. These results highlight the promising properties that Nb_2_O_5_-based coatings can provide to metallic materials for biomedical applications. In addition to the intriguing properties that niobium can offer, it is noteworthy that Brazil holds over 90% of the world’s niobium reserves. The state of Minas Gerais (MG) possesses the largest exploitable reserve in the country, with the Barreiro Mine, located in the city of Araxá, accounting for approximately 80% of global production [32]. Although Brazil already commercializes processed niobium in the form of iron–niobium (Fe-Nb) alloys and other products, it is essential to expand and promote the use of niobium.

This article delineates significant advancements in the utilization of Nb_2_O_5_ thin films deposited via reactive sputtering onto the surface of the Ti-6Al-4V alloy, with a particular emphasis on their potential applications within the biomedical sector. The study investigates the influence of Nb_2_O_5_ coatings on micro-wear properties under ambient air conditions and in AS solution.

## 2. Experimental

### 2.1. Research Plan

To achieve the objectives outlined in this study, Ti-6Al-4V samples were initially coated with Nb_2_O_5_ using the PVD technique, as detailed in the methodology presented by Machuno et al. [33]. Additionally, the morphology of the Ti-6Al-4V alloy surface was assessed both prior to and following the deposition process using SEM/EDX. The coating’s thickness and morphology were evaluated through atomic force microscopy (AFM) analysis. Furthermore, wear tests were conducted using a Pin-on-Disk apparatus in air and AS solution. Finally, the topography of the wear tracks was examined using SEM/EDX and profilometry analyses.

### 2.2. Material

In this study, Ti-6Al-4V specimens were used in their as-received state, possessing a chemical composition given in weight percentages of 6.75% Al, 4.5% V, 0.40% Fe, 0.08% C, 0.05% Ni, with the balance being titanium [34]. The properties of this alloy can vary considerably depending on the distribution of the two primary phases within its matrix. These phases are categorized as α and β, exhibiting hexagonal close-packed (HCP) and body-centred cubic (BCC) crystalline structures, respectively. To prepare the metallic substrate for coating application and to characterize the specific microstructure, the samples measured 1.5 mm × 1.5 mm × 2 mm underwent sanding with silicon carbide (SiC) sandpaper in the grit sizes of 800, 1200, 2400, and 4000#. Following the sanding process, the samples were cleaned for 15 min at room temperature using distilled water and isopropyl alcohol. The specimens were then securely stored in suitable holders until deposition using the reactive sputtering technique.

### 2.3. Nb_2_O_5_-Sputtered Coating Production and Morphological Characterization

The depositions were carried out in a reactive cathodic sputtering system, adhering to the guidelines outlined by Machuno et al. [33] at the Thin Film and Plasma Process Laboratory/Federal University of Triângulo Mineiro (UFTM), Uberaba, Minas Gerais State, Brazil. The film depositions involved careful consideration of the distance between the samples and the target, voltage, and current used in the DC power source, along with the deposition time and partial pressures of the gases. The target material used was niobium (99.999%), donated by Companhia Brasileira de Metalurgia e Mineração (CBMM). The chamber atmosphere consisted of a mixture of argon (99.999%) and O (99.999% from White Martins) at partial pressures of 5.0 mTorr and 0.5 mTorr, respectively, with an applied voltage of 440 V and a current of 140 mA. It is worth mentioning that a constant temperature of 300 °C in the PVD chamber was employed to promote greater film adhesion and homogeneity. Further details on the reactive sputtering process and the Nb_2_O_5_ film characterization can be found in the following references [5,30,31]. The surface topography and elemental composition of the samples, both with and without Nb_2_O_5_ coatings, were assessed using a field emission scanning electron microscope (FEG-SEM JEOL 7001F), and an EDX detector was coupled to the SEM in the Department of Physics at the Federal University of Paraná (UFPR), Curitiba, Paraná state, Brazil.

The surface morphology of the coated Ti-6Al-4V alloy was characterized using AFM at the Federal University of Triângulo Mineiro (UFTM), Uberaba, Minas Gerais state, Brazil. For this purpose, the AFM analyses were conducted using Shimadzu SPM 9700 equipment. AFM offered high-resolution imaging at the nanoscale, allowing for precise measurement of the Nb_2_O_5_ coating thickness in the present work by using the reactive sputtering technique.

### 2.4. Wear Tests

**Figure 1:**
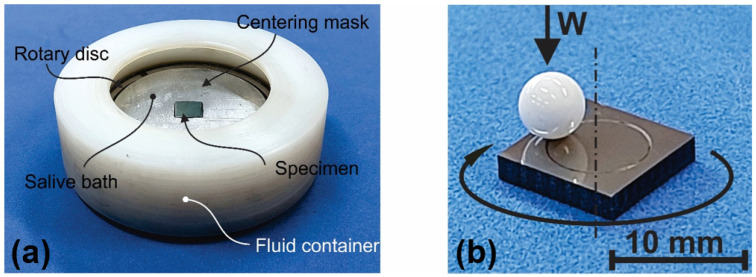
Wear test setup: (**a**) the assembled rotating support with the specimen immersed in AS, and (**b**) the specimen under the alumina ball showing a track after wear test.

The sliding wear behavior and coefficient of friction (COF) of the Ti-6Al-4V and Ti-6Al-4V/Nb_2_O_5_ specimens were assessed against a 4.78 mm alumina sphere as a counterpart in a Pin-on-Disk tribometer, following ASTM G99-05 [35]. The tests were conducted using two different ambient conditions. The first was performed without lubrication at a relative humidity of 50 to 60%, and the second with AS immersion (simulating oral conditions) at room temperature. The specimens were mounted on a rotating support with a surface load of 5 N applied to an off-center alumina sphere, resulting in a track diameter of 7 mm with a sliding speed of 0.01 m s^−1^ for 600 s sliding. Figure 1 shows a setup of the wear test: (a) the assembled rotating support with the specimen immersed in AS solution, and (b) the specimen under an alumina ball showing a track after testing. The alumina sphere (Macea) and the neutral pH AS (Santa Paula Pharmacy, Araraquara, São Paulo state, Brazil) were changed after each test. Table 1 displays the chemical composition of the AS solution used on the wear tests based on the work proposed by reference [36]. After the Pin-on-Disk test, the wear track analysis was performed by using a VEECO Wyko NT1100 non-contact optical profiler equipped with a 20× objective lens and a 0.5× field of view lens. Vertical scanning interferometry (VSI) was used, achieving a vertical resolution of <1 Å Ra and a lateral spatial sampling range of 0.08 to 13.1 μm. A consistent scanning area of 1.9 mm × 2.4 mm was used for all measurements. To enhance data reliability, four measurements were taken at 90° intervals around each wear track, with the average and standard deviation subsequently calculated. Additionally, parameters such as roughness and peak-to-valley distance were obtained considering different regions of the sample outside the wear track to evaluate the environmental influence on these parameters. Finally, the worn surface morphologies were evaluated by SEM, using an FEI Inspect S50 equipped with an EDX detector.

## 3. Results

**Figure 2:**
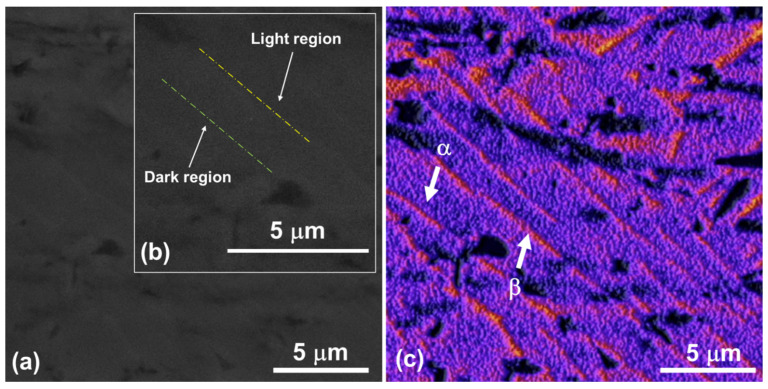
Surface SEM images of the Ti-6Al-4V alloy, (**a**) SE image with 1.0 kx magnification, (**b**) magnification of region showing light and dark regions, and (**c**) same image shown in (**a**) treated with the *ImageJ* software, showing the presence of α and β phases.

**Figure 3:**
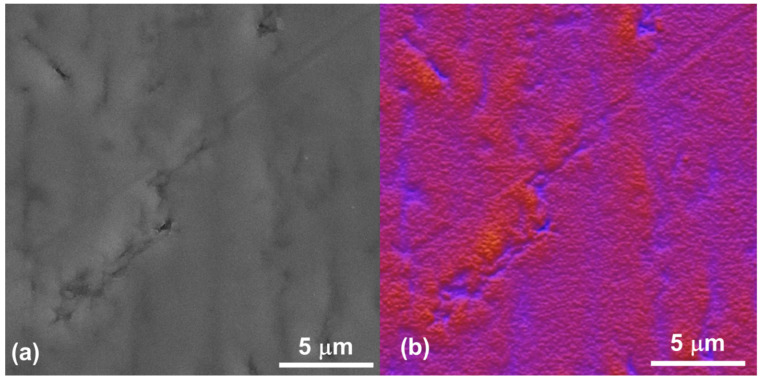
Surface SEM images of the Ti-6Al-4V alloy containing Nb_2_O_5_ thin film, (**a**) SE image with 1.0 kx magnification, and (**b**) same image shown in Figure 2a treated with the *ImageJ* software.

Figure 2a presents an SEM image of the Ti-6Al-4V alloy following the polishing procedure. Specifically, Figure 2b shows a secondary electron (SE) micrograph showing distinct light and dark regions, representing the α and β phases. The differing contrast between these regions reflects variations in material composition and/or crystallographic orientation. Figure 2c shows the same image as Figure 2a, but it is processed using *ImageJ* software (version 1.53) to enhance the visualization of the Ti-6Al-4V alloy’s morphology. Figure 3a displays the SEM image of the Ti-6Al-4V alloy containing Nb_2_O_5_ thin film obtained in the SE mode, whilst Figure 3b shows the treated image processed with *ImageJ* software. Both images revealed the presence of surface scratches resulting from the polishing procedure. These observations are supported by the AFM results presented below. The surface properties of biomedical implants are paramount to their long-term efficacy. Surface topography, chemistry, and wettability significantly influence protein adsorption, cell adhesion, and subsequent tissue integration. Crucially, these surface characteristics also govern bacterial adhesion and biofilm formation; rough surfaces generally provide increased sites for bacterial attachment, thereby increasing the risk of infection. Conversely, smooth surfaces, or those modified with specific chemistries, can minimize bacterial adhesion, creating a less conducive environment for bacterial colonization. Consequently, engineering-implanted surfaces reduce bacterial adhesion and promote osseointegration, which is vital for preventing implant failure and improving patient outcomes.

**Figure 4:**
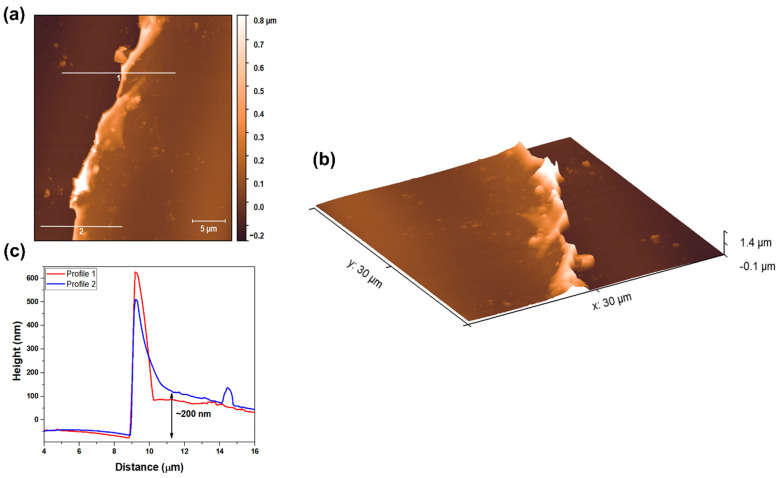
(**a**,**b**) Topographic surfaces of the Nb_2_O_5_ coatings deposited on commercial and polished glass substrates using the reactive sputtering technique, and (**c**) the profilometer values in two regions indicated by traces 1 and 2 in (**a**).

Figure 4 illustrates the results obtained from AFM in contact mode for Nb_2_O_5_ coatings deposited on commercial and polished glass substrates using the reactive sputtering technique. Figure 4a depicts the results obtained after removing the Kapton tape, which was employed to ascertain the thickness of the Nb_2_O_5_ coating at various points on the surface. It is apparent that some debris remains on the film surface due to the tape removal, despite subsequent cleaning with isopropanol, water, and a nitrogen jet. Two profiles were used to delineate the interface between the substrate and the Nb_2_O_5_ thin film, enabling an estimation of the film thickness, which was determined to be approximately 200 nm, as reflected in the graph presented in Figure 4c. The substrate–film interface is illustrated in three-dimensional topography in Figure 4b to elucidate the distinctions between the two layers. Using AFM images and Gwyddion software, the mean roughness (S_a_) of the Nb_2_O_5_ coating and the glass substrate was estimated at 15.3 nm and 10.0 nm, respectively. As the deposition process was conducted via reactive sputtering, an increase in film roughness relative to the glass substrate was observed, attributed to the nucleation process occurring in the initial stages of film formation.

**Figure 5:**
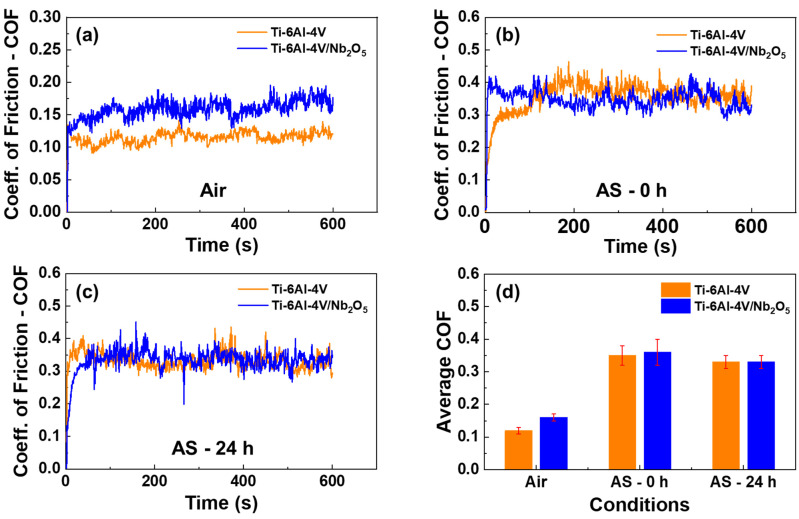
Coefficient of friction (COF) as a function of the environmental conditions for the Ti-6Al-4V and Ti-6Al-4V/Nb_2_O_5_ (**a**) in air after immersion on AS for (**b**) 0 h and (**c**) 24 h, and (**d**) average COF for all the tested samples as a function of the environmental conditions.

Figure 5 presents the COF behavior as a function of running time for Ti-6Al-4V and Ti-6Al-4V/Nb_2_O_5_ samples across all tested conditions. For both samples, the lower COF values were observed in tests conducted in the air (see Figure 5a), although the sample with the Nb_2_O_5_ film is slightly higher than the uncoated sample. For the analyses performed under immersion in AS for 0 and 24 h, the average coefficient of friction (COF_avg_) for both samples showed no significant differences, as depicted in Figure 5b,c. This can be attributed to the presence of the AS solution, which increases the contact between the sample surface and the Al_2_O_3_ sphere counterpart. Table 2 provides the calculated COF_avg_ values from the Pin-on-Disk tests.

**Figure 6:**
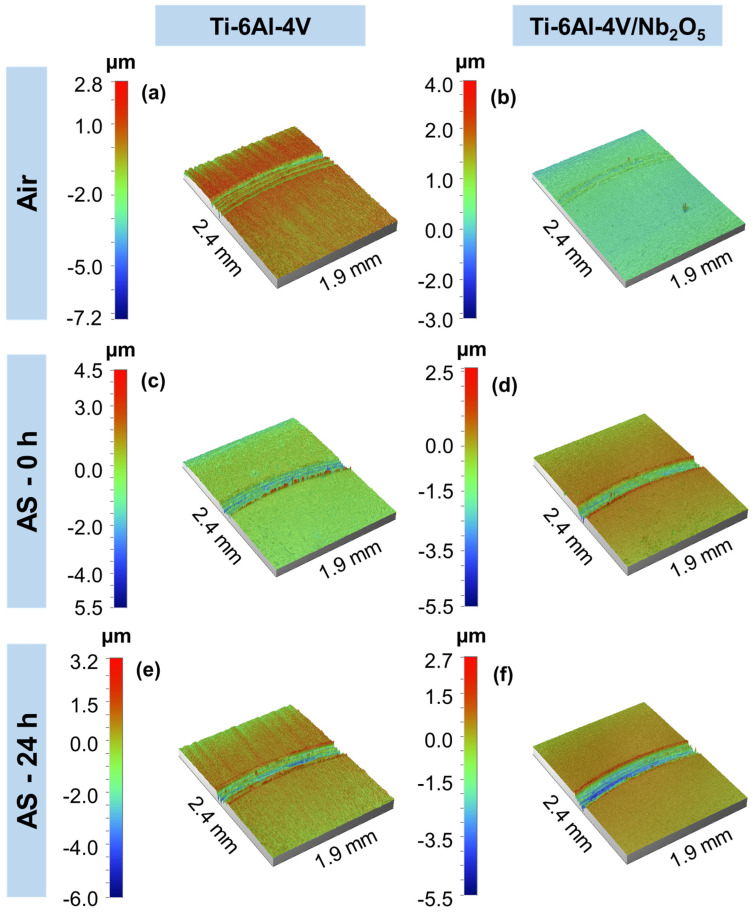
Profilometry analysis from the uncoated and coated Ti-6Al-4V (**a**,**b**) in air, after immersion on AS for (**c**,**d**) 0 h and (**e**,**f**) 24 h, respectively.

**Figure 7:**
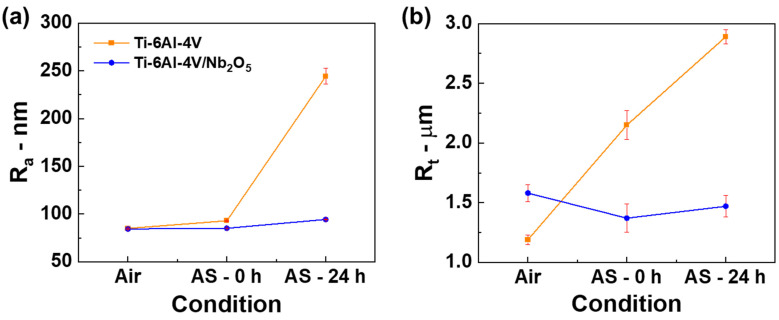
(**a**) R_a_ and (**b**) R_t_ values as a function of the environmental exposure condition considering the Ti-6Al-4V and Ti-6Al-4V/Nb_2_O_5_ samples.

To improve the assessment of the wear performance of both coated and uncoated specimens, the profiles of the wear tracks were analyzed using profilometry (refer to Figure 6). Under all testing conditions, the Ti-6Al-4V and Ti-6Al-4V/Nb_2_O_5_ samples exhibited an abrasive wear mechanism, which was characterized by parallel grooves aligned with the direction of wear. In samples tested in air, Figure 6a,b, the wear track for the Ti-6Al-4V/Nb_2_O_5_ sample is nearly imperceptible compared to the uncoated Ti-6Al-4V. For the samples tested immediately after AS immersion, Figure 6c,d both showed noticeable material displacement at the wear track border, with the base substrate presenting higher peak heights and a larger track diameter compared to the coated sample. Finally, Figure 6e,f illustrate the wear track profile for the Ti-6Al-4V and Ti-6Al-4V/Nb_2_O_5_ samples after 24 h of immersion on AS, where the coated sample exhibited a significantly reduced wear track diameter in comparison to the uncoated Ti-6Al-4V, suggesting the coating’s efficacy in protecting the metallic matrix against wear. In addition to the wear track profile obtained with the profilometry analysis, Figure 7 displays the average roughness (R_a_) and distance from peak-to-valley (R_t_) obtained for the uncoated and coated Ti-6Al-4V samples considering all the tested conditions. As expected, similar values of R_a_ and R_t_ were observed among both Ti-6Al-4V (84.96 ± 0.08 nm and 1.19 ± 0.04 µm, respectively) and Ti-6Al-4V/Nb_2_O_5_ (84.34 ± 0.56 nm and 1.58 ± 0.07 µm, respectively) considering the air environment, since the Nb_2_O_5_ thin film does not promote a significant modification on substrate topography due to its relatively low thickness, around 200 nm [3,5]. Regarding the samples after the Pin-on-Disk analysis under 0 h immersion in AS, since the exposure time in this aggressive environment is low, no significant modification in the R_a_ and R_t_ values is perceived, compared to air, for Ti-6Al-4V/Nb_2_O_5_ (85.07 ± 0.49 nm and 1.37 ± 0.12 µm) and a slight increase, around 10%, for the Ti-6Al-4V (92.93 ± 0.52 nm and 2.14 ± 0.12 µm, respectively) associated with the onset of corrosion processes in the metallic matrix. Finally, considering the samples after 24 h immersion in AS, the coated sample exhibited a 10% increment in the R_a_ and R_t_ values (94.39 ± 1.92 nm and 1.47 ± 0.09 µm, respectively), similar to the base material for 0 h immersion in AS. However, for the uncoated Ti-6Al-4V, after the 24 h immersion in AS, the values of R_a_ and R_t_ were, respectively, 224.23 ± 8.35 nm and 2.89 ± 0.06 µm, representing an increment of approximately 190% in comparison to the sample in air. This increase in the average roughness and peak–valley distance can be associated with the attack promoted by the aggressive medium on the metal matrix, which leads to the formation of corrosion products that, despite sealing the defects present on the TiO_2_ thin film, promote an intense modification on the substrate topography.

**Figure 8:**
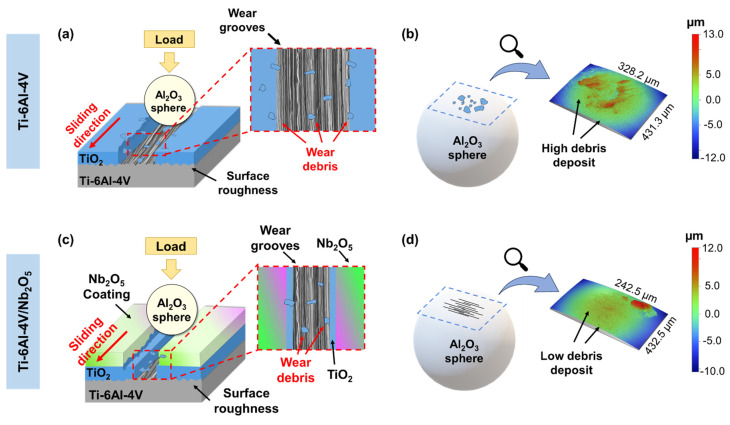
Wear mechanism representation for the Pin-on-Disk analysis in air and profilometry images for the respective spheres employed, being (**a**,**b**) Ti-6Al-4V and (**c**,**d**) Ti-6Al-4V/Nb_2_O_5_.

To gain a deeper insight into the interaction between the spheres and the coated and uncoated Ti-6Al-4V samples, supplementary profilometry analyses of the Al_2_O_3_ sphere counterparts were performed. Figure 8 presents a schematic representation of the wear mechanism for Ti-6Al-4V and Ti-6Al-4V/Nb_2_O_5_ specimens in the air environment, along with profile images of the interaction region on the Al_2_O_3_ spheres’ surfaces after the Pin-on-Disk test. When comparing both samples tested in air, a difference in the wear track diameter is observed, attributed to the modification of the surface hardening promoted by the Nb_2_O_5_ coating and the respective wear mechanisms in each case (Figure 8a,c). Comparing the spheres, the one in contact with the uncoated sample displayed more debris and grooves on its surface (Figure 8b), indicating a more intense interfacial interaction and material removal from the sample compared to Ti-6Al-4V/Nb_2_O_5_ (Figure 8d). Additionally, it is worth mentioning that the spheres used in the Pin-on-Disk analysis presented average R_a_ and R_t_ values of 37.91 ± 8.45 nm and 1.54 ± 0.26 µm, respectively.

**Figure 9:**
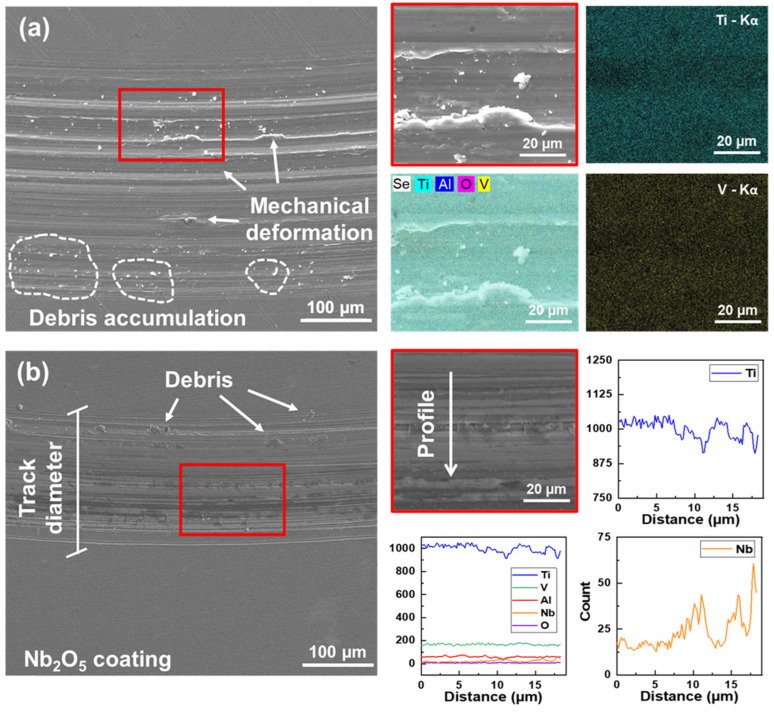
SEM/EDX wear track analysis for both (**a**) Ti-6Al-4V and (**b**) Ti-6Al-4V/Nb_2_O_5_ tested in air.

**Figure 10:**
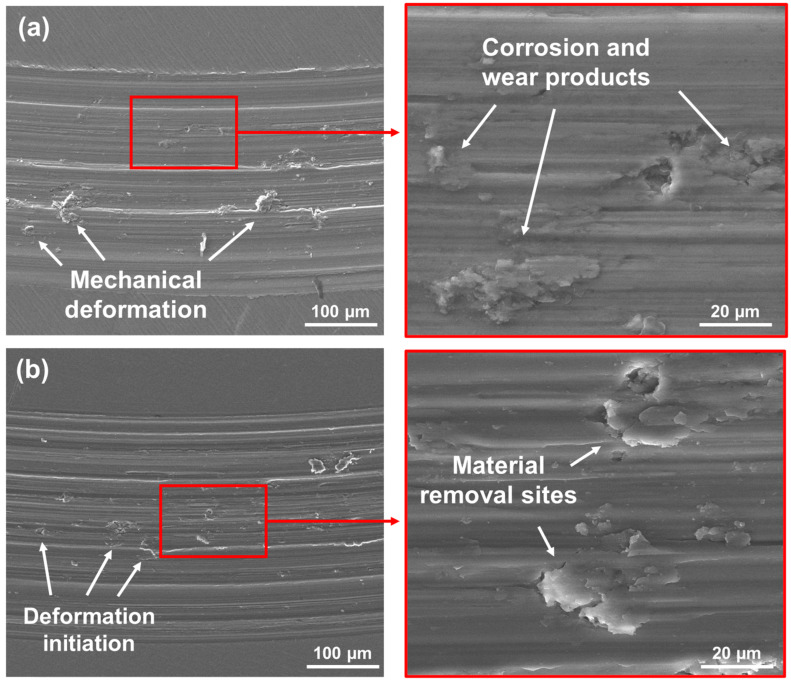
SEM/EDX wear track analysis for both (**a**) Ti-6Al-4V and (**b**) Ti-6Al-4V/Nb_2_O_5_ after 24 h of immersion in AS solution.

Figure 9 and Figure 10 present a schematic assembly of the SEM/EDX analysis for the Ti-6Al-4V and Ti-6Al-4V/Nb_2_O_5_. As confirmed by the SEM micrographs, several debris and plastic mechanical deformations can be observed along the wear track profile for the uncoated sample in air, which is associated with a more severe material removal, Figure 9a. However, the coated Ti-6Al-4V alloy exhibited a more preserved surface, with a low amount of abrasive mass loss and, as verified by EDX analysis, the presence of remaining Nb_2_O_5_ thin film, Figure 9b. Finally, considering the samples tested after 24 h of immersion in AS, the uncoated sample presented not only the wear particles that were observed after the air analysis but also cavities and deposited compounds with small quantities of Cl, indicating the possible formation of corrosion products, Figure 10a. For the Ti-6Al-4V/Nb_2_O_5_ sample after 24 h immersion in AS, removal of the coating was verified along with initial signs of plastic mechanical deformation within the wear track, Figure 10b. Moreover, it is worth mentioning that the chemical composition of the debris particles inside the wear track was analyzed by EDX, being composed of O 5.78%, Al 5.22%, V 1.95% and Ti as balance (% in weight), which indicates a slightly higher value of O content in comparison to the Ti-6Al-4V matrix (O 2.38%, Al 4.42%, V 2.46% and Ti as balance). The difference in the O values is expected and associated with a TiO_2_ layer formation around the particles due to the increased contact surface.

**Figure 11:**
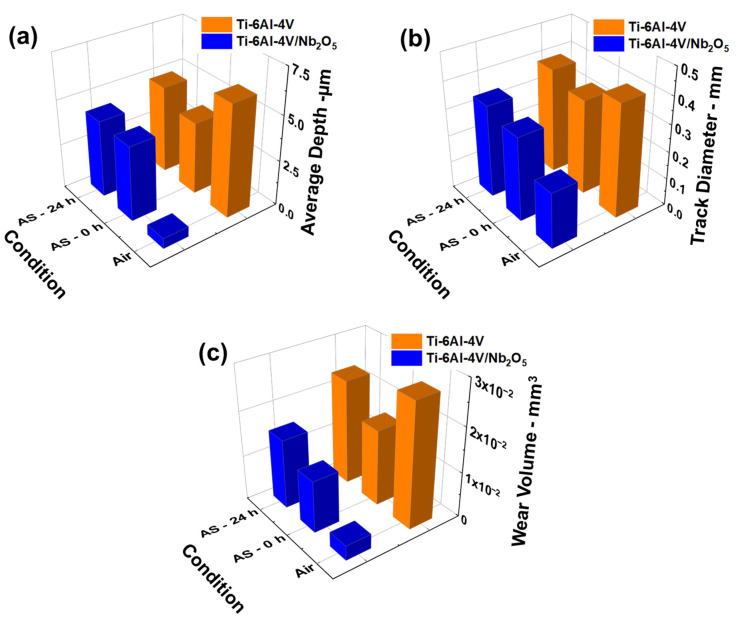
Complementary data from the wear track profile from the Ti-6Al-4V and Ti-6Al-4V/Nb_2_O_5_ samples obtained from the profilometry analysis, (**a**) average track depth, (**b**) wear track diameter, and (**c**) wear volume.

For a quantitative assessment of the wear performance of the tested samples, the profilometry analysis yielded critical parameters in conjunction with the profile maps previously presented. Figure 11a–c illustrate data regarding the average depth of the wear tracks, the track diameter, and the calculated wear volume based on the ASTM G99-05 specifications for each environmental condition [35]. Notably, the ratio of the sphere radius to the wear track diameter remains below 0.1 for all samples tested, resulting in an estimated calculation error of approximately 1%, considering the ASTM standard description. Furthermore, Table 3 details all profilometry data obtained for the Ti-6Al-4V and Ti-6Al-4V/Nb_2_O_5_ samples across the various tested conditions.

## 4. Discussion

Regarding the wear test on the Pin-on-Disk tribometer, the Ti-6Al-4V samples tested in air exhibited a slightly lower average COF than those from the Ti-6Al-4V/Nb_2_O_5_. Although the COF reflects the interaction dynamics between two surfaces, a higher value does not necessarily correspond to a higher wear process since it depends on surface parameters such as hardness and the environment in which the material is inserted [37,38,39,40]. This was substantiated by profilometry analysis, which showed that the Nb_2_O_5_ coating significantly reduced both the depth and diameter of the wear track, as well as decreased the wear volume by approximately 88% in comparison to the uncoated material. Furthermore, the SEM/EDX results for the coated sample indicated that, even after 600 s of testing in the Pin-on-Disk device in air, the Nb_2_O_5_ film remained present inside the wear track (Figure 9b). This demonstrates that the wear occurred mainly on the coating, with the metal substrate being protected against degradation. Mercer and Hutchings [41] explored the effect of various atmospheric conditions on the abrasive wear behavior of pure Ti and Ti-6Al-4V alloys using a Pin-on-Disk system. As pointed out by the authors, the high reactivity of Ti with elements such as O in the atmosphere leads to the constant formation of the TiO_2_ thin film on the material surface during the wear tests. This constant repassivation facilitates the debris removal from the track, intensifying the wear process on the sample surface by limiting the accumulation of particles that act as lubricants (Figure 8a). Moreover, this hypothesis is supported by observations of a more extensively worn sphere surface with the accumulation of debris as well as a greater amount of removed material retained within the track and the presence of marks indicative of intense plastic mechanical deformation in the abrasive path after the wear test in air on the Ti-6Al-4V sample, see Figure 8b,d. Since the coated sample’s surface is partially isolated from atmospheric exposure, the TiO_2_ formation is limited, see Figure 8c. Consequently, surface hardness becomes the dominant factor influencing wear behavior in both coated and uncoated Ti-6Al-4V samples. These results are in accordance with those observed in different works in the literature [42,43,44].

**Figure 12:**
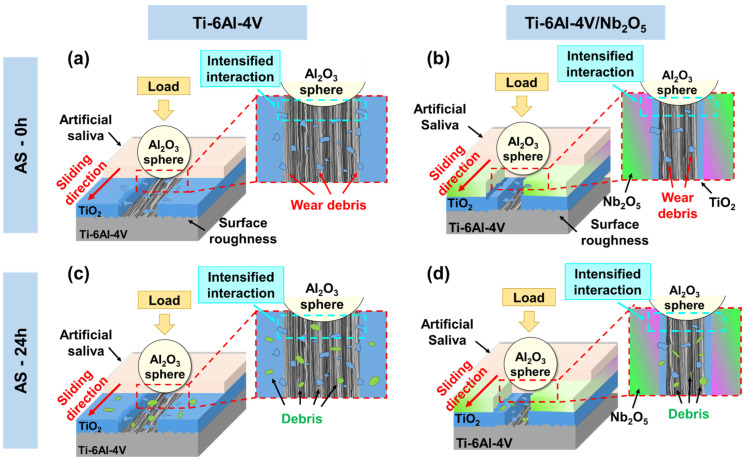
Schematic representation of the wear mechanism associated with the (**a**,**c**) Ti-6Al-4V and (**b**,**d**) Ti-6Al-4V/Nb_2_O_5_ samples considering the different times of immersion on AS.

In analyzing the effect of varying exposure times to an AS environment, it is evident that immersion in this medium introduces complexity to the wear mechanisms. Figure 12 presents a schematic representation of the mechanisms associated with the presence of an aggressive medium on the wear performance of the Ti-6Al-4V and Ti-6Al-4V/Nb_2_O_5_. Both coated and uncoated samples exhibited an increased COF, reaching approximately 0.35; this increase is likely attributable to the viscosity of the AS, which, rather than acting as a lubricant, intensified the contact between the sphere and the sample surfaces, Figure 12a,b. However, as previously mentioned, the COF is only a primary indication of the material wear tendency, and the actual wear mechanism is influenced by several other factors [45,46]. For the analysis considering 0 h of immersion in AS, the R_a_ and R_t_ values remained similar for both the Ti-6Al-4V and Ti-6Al-4V/Nb_2_O_5_, which is expected due to the low samples-environment interaction, despite the slight increase of 10% for the base material in comparison to the samples tested in air (92.93 ± 0.52 nm and 2.14 ± 0.12 µm, versus 85.07 ± 0.49 nm and 1.37 ± 0.12 µm, respectively). The uncoated Ti-6Al-4V alloy demonstrated a reduced wear volume compared to those tested in air (1.69 × 10^−2^ ± 0.16 × 10^−2^ and 2.77 × 10^−2^ ± 0.32 × 10^−2^ mm^3^, respectively) despite the increase on the COF, representing a 39% reduction. This phenomenon can be explained by the limited formation of TiO_2_ due to the reduced availability of surface O previously mentioned [41]. Consequently, wear debris is retained within the wear track, thereby limiting material removal and mitigating wear processes. In contrast, the coated sample exhibited a slight increase in wear volume when tested in AS compared to air, a change attributed to the heightened sphere-surface interaction facilitated by the environment (1.16 × 10^−2^ ± 0.12 × 10^−2^ and 3.32 × 10^−3^ ± 0.11 × 10^−3^ mm^3^, respectively, for 0 h immersion in AS and air, respectively). Nevertheless, despite the increase in wear volume of the coated sample, the protective effect of the Nb_2_O_5_ thin film against wear in Ti-6Al-4V remains significant. Even under adverse conditions, the thin film continues to reduce wear volume by approximately 31% compared to the uncoated sample.

In scenarios involving prolonged exposure, the protective properties of the Nb_2_O_5_ thin film on Ti-6Al-4V become more pronounced, Figure 12c,d. After 24 h of immersion in AS, the R_a_ and R_t_ values (224.23 ± 8.35 nm and 2.89 ± 0.06 µm) of the uncoated surface increased markedly, attributed to the formation of corrosion products in brittle regions of the TiO_2_ layer and initiated a d-glucose film due to saccharides present in AS. As reported in the literature, with the immersion time advance in an aggressive environment, the Ti-base alloys show improved electrochemical behavior due to the electroactive sealing processes by the corrosion product formation [5,47]. However, while this improvement in anti-corrosion performance is achieved, a modification in the substrate topography is observed. This alteration directly affects the material’s wear performance. Additionally, in a previous study, the research group of Moreto and colleagues [48] evaluated the influence of environmental conditions on the electrochemical behavior of CoCrMo alloy used for implantable devices. Overall, two environments were analyzed, with both being an aerated physiological serum and Hank’s solution for temperatures of 25 and 37 °C, with exposure times of 5 min and 168 h; as indicated by the authors, an increase in the semi-capacitive arc on the Nyquist diagrams was observed for the CoCrMo alloy samples when comparing the exposure times of 5 min and 168 h in the presence of Hank’s solution. Moreover, considering the samples exposed to physiological serum solution, an opposite behavior was observed: as the immersion time increases, the corrosion resistance of the CoCrMo alloy decreases. Additionally, by using equivalent circuits for the electrochemical impedance spectroscopy data treatment, a significant reduction in the capacitance values associated with the Cr_2_O_3_ layer formed on the surface of this material was verified. This diminishment can be associated with a D-glucose film formation and subsequent ion adsorption instability on the metallic substrate, which reduces the electroactive area and enhances the CoCrMo alloy corrosion resistance.

The modifications on the surface properties of the Ti-6Al-4V, accompanied by an intensification process on the surface interaction due to the presence of the AS during the Pin-on-Disk analysis, result in an increase in the wear volume as the immersion time increases. On the other hand, the Ti-6Al-4V/Nb_2_O_5_ sample exhibited the same values of R_a_ and R_t_ as those tested in air (94.39 ± 1.92 nm and 1.47 ± 0.09 µm, respectively), despite a slight 10% increase, indicating that the thin film not only protected the matrix against corrosion but also mitigated the formation of such layer without changing the surface properties of the substrate. It is crucial to emphasize that the Nb_2_O_5_ thin film demonstrated stability even after prolonged immersion in various aggressive solutions. Additionally, it effectively enhanced the corrosion resistance of different metallic materials [5,30]. Moreover, when evaluating the effect of these films on the wear performance of the Ti-6Al-4V alloys, a significant reduction in the wear volume and wear rate was observed, consolidating these types of coatings as promising candidates to be applied as a first-layer protection for different materials within the biomedical industry [31].

Furthermore, the maintenance of these properties is highly desirable once they affect the interaction of the implantable device with the buccal environment, in which a modification can cause bacterial proliferation and inflammatory processes in the surrounding body tissue [35]. Moreover, this protective effect of the Nb_2_O_5_ thin film is summarized by a reduction of approximately 41% in the wear volume in comparison to the uncoated sample. In fact, even though the Ti-6Al-4V/Nb_2_O_5_ showed a slight rise in wear volume between 0 and 24 h of immersion in AS, the bare Ti-6Al-4V demonstrated a much more significant increase in its wear volume for the same period. In addition, the wear reduction promoted by the thin film deposited by using the PVD technique is comparable to other surface treatments presented in different studies in the literature [49,50]. Weng and colleagues [50] evaluated the influence of laser cladding coating process parameters on the wear performance of the Ti-6Al-4V alloy. As verified by the authors, a reduction of approximately 90% in mass loss was obtained by optimizing the laser cladding process. However, this improvement in the wear properties is associated with a modification of the phases present in the surface layers of the metal matrix, an effect inherent to the laser treatment process, improving the hardness properties and subsequent modification in its topography. These findings underscore the positive effect of the Nb_2_O_5_ thin films on protecting the metallic substrate against wear and mimicking the Ti-6Al-4V surface properties even for a long-term implantable device, as well as improving its performance against corrosion. Thus, consolidating the PVD technique as a promising alternative for surface functionalization that offers both low costs that can be industrially scaled and meaningful increment in the surface properties of different materials.

## 5. Conclusions

This study showed enhanced wear performance of the Ti-6Al-4V alloy with Nb_2_O_5_ coating in both air and AS. The test specimens were placed under alumina spheres and presented a consistent COF for both Ti-6Al-4V and Ti-6Al-4V/Nb2O5 surfaces. However, the coated specimens demonstrated lower wear rates in both environments. The reduction in wear can be attributed to the shift from adhesive wear mechanisms on uncoated surfaces to abrasive mechanisms on coated surfaces. These results indicate an improvement, as the coating enhances wear resistance, contributes to corrosion resistance, and improves biocompatibility. Regarding the results obtained in this study, the coating does not influence friction for certain products, such as screws, that require a high coefficient of friction.

## Figures and Tables

**Table 1:** Chemical composition of the AS solution used in the present work for the wear tests.

Chemical Composition	% Weight
Glucitol	20
AS concentrate	10
CMC (Thickener)	0.3
Phenochem+	0.2
Deionized water	Balance

**Table 2:** COF_avg_ for the Ti-6Al-4V and Ti-6Al-4V/Nb_2_O_5_, considering the different tested environmental conditions.

Conditions	Ti-6Al-4V			Ti-6Al-4V/Nb_2_O_5_		
**Air**	0.12	±	0.01	0.16	±	0.01
**AS-0 h**	0.35	±	0.03	0.36	±	0.04
**AS-24 h**	0.33	±	0.02	0.33	±	0.03

**Table 3:** Wear track depth, track diameter, and wear volume for the Ti-6Al-4V and Ti-6Al-4V/Nb_2_O_5_ samples considering all tested environmental conditions obtained by the profilometry analysis.

Condition	Sample	Track Depth	Track Width	Wear Volume
		(µm)	(mm)	(mm^3^)
**Air**	Ti-6Al-4V	6.21 ± 0.87	0.42 ± 0.02	2.77 × 10^−2^ ± 0.32 × 10^−2^
	Ti-6Al-4V/Nb_2_O_5_	0.58 ± 0.19	0.21 ± 0.01	3.32 × 10^−3^ ± 0.11 × 10^−3^
**AS—0 h**	Ti-6Al-4V	4.01 ± 0.56	0.35 ± 0.01	1.69 × 10^−2^ ± 0.16 × 10^−2^
	Ti-6Al-4V/Nb_2_O_5_	4.61 ± 0.18	0.31 ± 0.01	1.16 × 10^−2^ ± 0.12 × 10^−2^
**AS—24 h**	Ti-6Al-4V	4.87 ± 0.39	0.39 ± 0.02	2.34 × 10^−2^ ± 0.30 × 10^−2^
	Ti-6Al-4V/Nb_2_O_5_	4.27 ± 0.38	0.34 ± 0.01	1.54 × 10^−2^ ± 0.18 × 10^−2^

## Data Availability

The original contributions presented in this study are included in the article. Further inquiries can be directed to the corresponding author.
